# Interdevice variability of central corneal thickness measurement

**DOI:** 10.1371/journal.pone.0203884

**Published:** 2018-09-13

**Authors:** Peter M. Maloca, Harald P. Studer, Renato Ambrósio, David Goldblum, Simon Rothenbuehler, Daniel Barthelmes, Sandrine Zweifel, Hendrik P. N. Scholl, Konstantinos Balaskas, Adnan Tufail, Pascal W. Hasler

**Affiliations:** 1 OCTlab, Department of Ophthalmology, University Hospital Basel, Basel, Switzerland; 2 Department of Ophthalmology, University of Basel, Basel, Switzerland; 3 Institute of Molecular and Clinical Ophthalmology Basel (IOB), Basel, Switzerland; 4 Moorfields Eye Hospital, London, United Kingdom; 5 Swiss Eye Research Foundation, Reinach, Switzerland; 6 Department for Ophthalmology, Federal University of Sao Paulo, Sao Paulo, Brazil; 7 Rio de Janeiro Corneal Topography and Biomechanics Study Group, Rio de Janeiro, Brazil; 8 Instituto de Olhos Renato Ambrósio, Rio de Janeiro, Brazil; 9 Department of Ophthalmology, University Hospital Zurich, Switzerland; 10 Wilmer Eye Institute, Johns Hopkins University, Baltimore, United States of America; 11 Moorfields Ophthalmic Reading Centre, London, United Kingdom; University of Alabama at Birmingham School of Medicine, UNITED STATES

## Abstract

**Purpose:**

To evaluate variability of central corneal thickness measurement (CCT) devices using a hitherto unprecedented number of CCT devices.

**Methods:**

CCT was measured consecutively in 122 normal corneas of 61 subjects with seven different devices using three distinct measurement technologies: Scheimpflug, Ultrasound, and Optical Coherence Tomography (OCT). Per device deviation from the mean CCT value per eye was used to determine which of the devices performed best, compared to the mean value.

**Results:**

Cirrus OCT yielded the lowest deviation. Deviations of the individual devices from the mean CCT of each eye were (OS/OD) 12.8±5.0/14.9±9.4 μm for Topcon noncontact specular microscopy (NCSM), 11.3±5.9/10.6±7.3 μm for Pentacam, 10.7±5.2/10.4±4.8 μm for Spectralis OCT, 6.0±3.9/6.2±4.9 μm for Topcon DRI OCT, 5.1±3.4/5.9±10.3 μm for AngioVue OCT, 4.8±4.1/5.7±4.6 μm for US pachymetry, and 4.2±3.2/5.7±4.6 μm for Cirrus OCT. The maximum differences between US pachymetry and the other devices were very high (up to 120 μm).

**Conclusion:**

Central corneal thickness may be under- or overestimated due to high interdevice variations. Measuring CCT with one device only may lead to inappropriate clinical and surgical recommendations. OCT showed superior results.

## Introduction

Clinical decision making, whether for the Laser-in-situ-Keratomileusis (LASIK), or for monitoring and screening of various diseases, often relies on the results of CCT measurement. It has been found, that a thinner central corneal thickness (CCT) may represent a higher risk in primary open-angle glaucoma, and patients with a thinner CCT tend to exhibit a more severe glaucomatous defect. [1, 2]

CCT measurements helped diagnose subclinical keratoconus [3], identify contact-lens-induced corneal thinning [4], and monitor various corneal pathologies. [5, 6] Preoperative pachymetry assessment has been shown to be effective to avoid postsurgical corneal ectasia [7].Due to the high reproducibility for measuring CCT Ultrasound (US) pachymetry is still widely used and considered the gold-standard for CCT assessment. [8]

However, Doughty & Zaman reported that ultrasonic measurements tend to overestimate the cornea thickness than optical measurements. [9] Rotating Scheimpflug cameras have proven to be valuable tools, although the CCT values obtained from them have been shown to be slightly lower than those measured by US pachymetry. [10] Both Scheimpflug imaging and spectral-domain OCT (SDOCT) have been demonstrated with high precision, while the SDOCT possesses a higher repeatability. [11] Noncontact specular microscopy (NCSM) can image the corneal endothelial morphology, but underestimates CCT compared to US pachymetry. [12, 13]

Swept-source OCT (SSOCT) has shown a higher reliability than Scheimpflug imaging and allows for monitoring corneal thickness after photorefractive keratectomy. [14, 15]

To improve surgical procedures or treatment, this study compares hitherto unprecedented number of devices for central corneal thickness measurements. From seven (7) devices we calculated the lowest deviation from the mean CCT value.

## Materials and methods

### Subjects

Subject age, race, sex and diurnal fluctuations were not considered in this study as our goal was to collect the most possible CCT data from many devices in short time.

Sixty-one subjects who were visiting the clinic were randomly selected for CCT measurements using seven different devices. Inclusion criteria were as follows: Subjects with normal visual acuity (20/20), good and steady fixation, and clear cornea. Exclusion criteria included a history of corneal refractive surgery, corneal scarring, degenerations, and dystrophies. Written informed consent was obtained from all subjects, and the study was approved by the Ethics Committee Zentral und Ostschweiz, Switzerland (EKNZ UBE-15/881).

### Data acquisition

The order of measurements using different devices was random, apart from the US pachymetry that was always done last in order not to disturb the corneal surface and impair the non-contact measurements. All 14 measurements were conducted at the University of Basel, Switzerland, within a timeframe of 60 minutes. It was proposed to use central thickness measurements to increase comparability between devices. CCT was measured using the following seven devices: Oculus Pentacam version 1.19r11 (Oculus, Wetzlar, Germany), noncontact specular microscopy SP-1P version 1.21 (NCSM, Topcon, Tokyo, Japan), spectral-domain OCT with Cirrus HD-OCT 5000–2997 version 7.0 (SDOCT, Carl Zeiss Meditec, Dublin, CA, USA), Spectralis HRA 2 version 6.0.10.0 (Heidelberg Engineering, Heidelberg Germany), AngioVue OCT software version 2014.2.0.93 (Optovue, Inc., Fremont, CA, USA), swept-source OCT with prototype OCT DRI OCT-1 Atlantis software version 9.12.003.04 (SSOCT, Topcon, Tokyo, Japan), and US pachymetry ALCON RxP version 1.15 (Alcon, Fort Worth, TX, USA).

Automatic acquisition mode was used for the rotating Scheimpflug camera and for NSCM to capture CCT. For SDOCT, additional anterior lens systems were used in the Spectralis and AngioVue devices. The OCT scan patterns were: Cirrus anterior segment 5-line raster scan protocol without anterior segment lens (scan length 3 mm, interslice distance 0.25 mm, 4,096 A-scans per line), Spectralis anterior segment protocol with anterior segment lens (21 B-scans, scan area 8.3 mm x 5.6 mm, interslice distance 278 μm), AngioVue cornea pachymetry protocol with anterior segment lens (scan area 6 mm x 6 mm, measurement within central 3 mm zone), and Topcon SSOCT volume scan patterns over a 6.0 mm x 6.0 mm area centered on the corneal apex (256 cross scans with a scan density of 512 x 128 pixel). The manufacturer caliper tools were used to measure the CCT. The US pachymetry measurement of the cornea was performed as the last test a few minutes after the local application of preservative-free oxybuprocaine (0.4%). Three consecutive measurements were taken from the central cornea. For all measurements the thinnest corneal point was selected.

The axial resolution is 1μm for both the ultrasound device Alcon RxP [16] and for the Pentacam [17], 2 to 3μm for the AngioVue [18], 10μm for the NCSM [19], 3.5μm for the Heidelberg Spectralis [20], 5μm for the Cirrus HD-OCT [21], and 8μm OCT DRI OCT-1 Atlantis [22].

### Statistical analysis

Statistical analysis was performed using Microsoft Office Excel 2013 (Microsoft Corp., Redmond, WA). Analysis of variance (ANOVA) and Chi-Square(Х2)-test were performed but not included in this manuscript as this study represents a method comparison study ranking pairs of methods on the basis of their extent of agreement. There were seven groups (*G*_*i*_) of data, one group for each measurement device. Each group *G*_*i*_ thereby consists of 61 data points, one point per eye. Our statistical analysis assumes that the value of each of these 61 data points *G*_*i*,*n*_ corresponds to the following sum: the device-independent true CCT value *t*_*n*_ of the eye, the device-dependent bias *b*_*i*_, and the device-dependent measurement error *e*_*i*_:

$$G_{i,n}=t_{n}+b_{i}+e_{i},$$ (1)

where the error *e*_*i*_ is assumed to have a normal distribution *N*(0,*v*_*i*_). In order to determine a possible between-group difference, paired differences of Eq (1) were formed as follows

$$D_{i}=G_{i}-G_{j}=\{b_{i}-b_{j}\}+\{e_{i}-e_{j}\},$$ (2)

where *i*,*j* ∈ (Pentacam, NSCM, US, Cirrus, AngioVue, Spectralis, and DRI) and *i* ≠ *j*. For un-replicated data, the mean and standard deviation of the between-group differences *D*_*i*_ usually convey the most useful information. Paired device-by-device differences between US pachymetry and the other six devices were computed and analyzed. As a summary statistic of inter-device variation, the mean absolute deviation from a central point, the mean CCT over all seven devices per eye, was calculated as

$$v_{i,k}=\frac{1}{7}\sum_{i=1}^{7}\left|x_{i,k}-m(X)\right|,$$ (3)

where *x*_*i*,*k*_ are the 7 measurements (*i* ∈ Pentacam, NSCM, US, Cirrus, AngioVue, Spectralis, and DRI) for the *k*-th eye (one measurement value per device), and *m*(*X*) is the mean of the 7 values, to investigate deviation of the measurements. Mean and standard deviation, calculated as

$$m_{i}=\frac{1}{n}\sum_{k=1}^{n}v_{i,k},$$ (4)

$${SD}_{i}=\frac{1}{n}\sum_{k=1}^{n}{\left(v_{i,k}-m_{i}\right)}^{2},$$ (5)

express the deviation of each individual device *i*.

## Results

This study measured the central corneal thickness in 122 normal eyes from 61 subjects with an average age of 63.7 years (range 20–86 years). No study subject was excluded. Men represented 34% of the study subjects (66% females).

**Fig 1:**
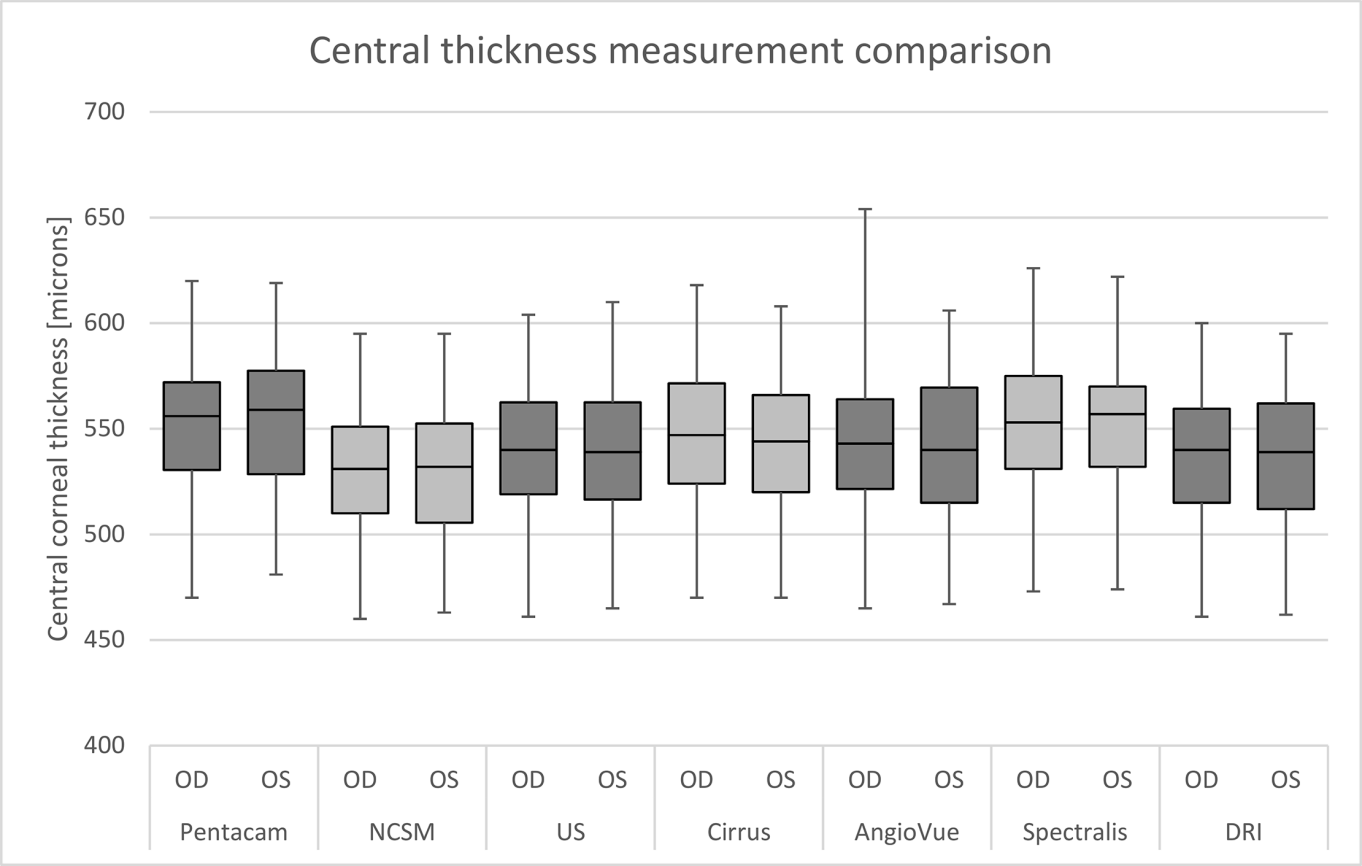
Comparison of central corneal thickness (CCT) measurements. Significant differences between devices are illustrated (Pentacam/US p<0.0001, Topcon/US p<0.0001, Cirrus/US p<0.0001, AngioVue/US p = 0.092, Spectralis/US p<0.0001, and DRI/US p = 0.004). However, no significant difference was found between left and right eyes (Pentacam p = 0.687, Topcon p = 0.864, Cirrus p = 0.745, AngioVue p = 0.709, Spectralis p = 0.973, DRI p = 0.955, and US pachymetry p = 0.866).

The overall mean CCT values were as follows (OS/OD): Pentacam 552±35/550±34 μm (SD), Topcon 529±34/530±30 μm (SD), Cirrus 544±34/546±33 μm (SD), AngioVue 540±32/540±32 μm (SD), Spectralis 552±34/553±35 μm (SD), DRI 537±34/537±33 μm (SD), and US pachymetry 539±35/539±36 μm (SD), (Fig 1).

CCT ranges measured by different devices in the left eyes are Pentacam 481–619 μm, Topcon 463–595 μm, Cirrus 470–608 μm, AngioVue 467–606 μm, Spectralis 474–622 μm, DRI 462–595 μm, and US pachymetry 465–610 μm.

CCT ranges measured by different devices in the right eyes are Pentacam 470–620 μm, Topcon 460–595 μm, Cirrus 470–618 μm, AngioVue 465–654 μm, Spectralis 473–626 μm, DRI 461–600 μm, and US pachymetry 461–604 μm.

### Between-device differences

**Fig 2:**
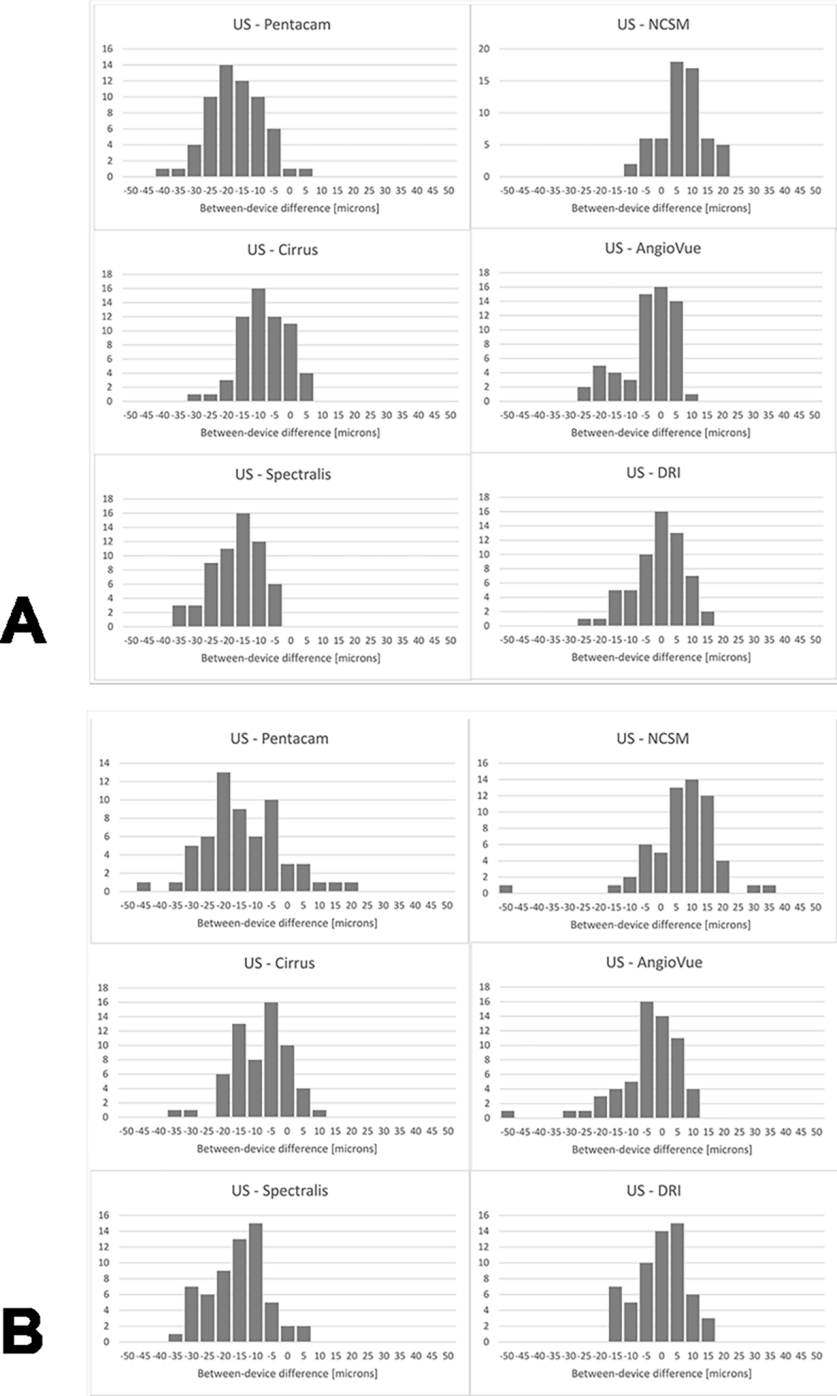
Histogram representations of paired between-device differences of the left eyes (2. A) and right eyes (2. B). Compared to the ultrasound device, mean and standard deviation difference clearly exist between devices. While some devices over-estimate CCT in average (e.g. Pentacam), other under-estimate it (e.g. NCSM). Furthermore, while some comparisons show rather large standard deviation (e.g. Pentacam), some match better with the US device (e.g. DRI).

Between-device differences *D*_*i*_ were analyzed by looking at the mean and standard deviation of paired CCT difference values between each device and the US pachymetry device (OS/OD). A “-”indicates that a device measured thicker, and a “+” indicates that a device measured thinner CCT values than the ultrasound device: US-Pentacam -14.5±7.8/-10.9±12.5 micron (SD), US-NSCM +9.4±8.0/+9.5±15.6 micron, US-Cirrus -5.3±7.5/-6.2±8.9 micron, US-AngioVue -1.3±8.9/-2.6±15.3 micron, US-Spectralis -14.1±7.6/12.8±8.9 micron, and US-DRI +1.9±8.6/+2.6±8.5 micron. These values and the histogram representations in Fig 2 show that Pentacam overestimates CCT with respect to US pachymetry.

NCSM is similar to US pachymetry for small values, but underestimates higher values. Cirrus slightly overestimates lower values with respect to US pachymetry. For higher values, the two devices are in good agreement. AngioVue overestimates low values and underestimates higher values with respect to US pachymetry. Spectralis overestimates low values and is in good agreement with US pachymetry for higher values. DRI SSOCT overestimates low and underestimates higher values with respect to US pachymetry. Even more surprisingly, there are differences of up to 120 μm between US pachymetry and the NCSM device.

### Correlation and agreement between devices

**Fig 3:**
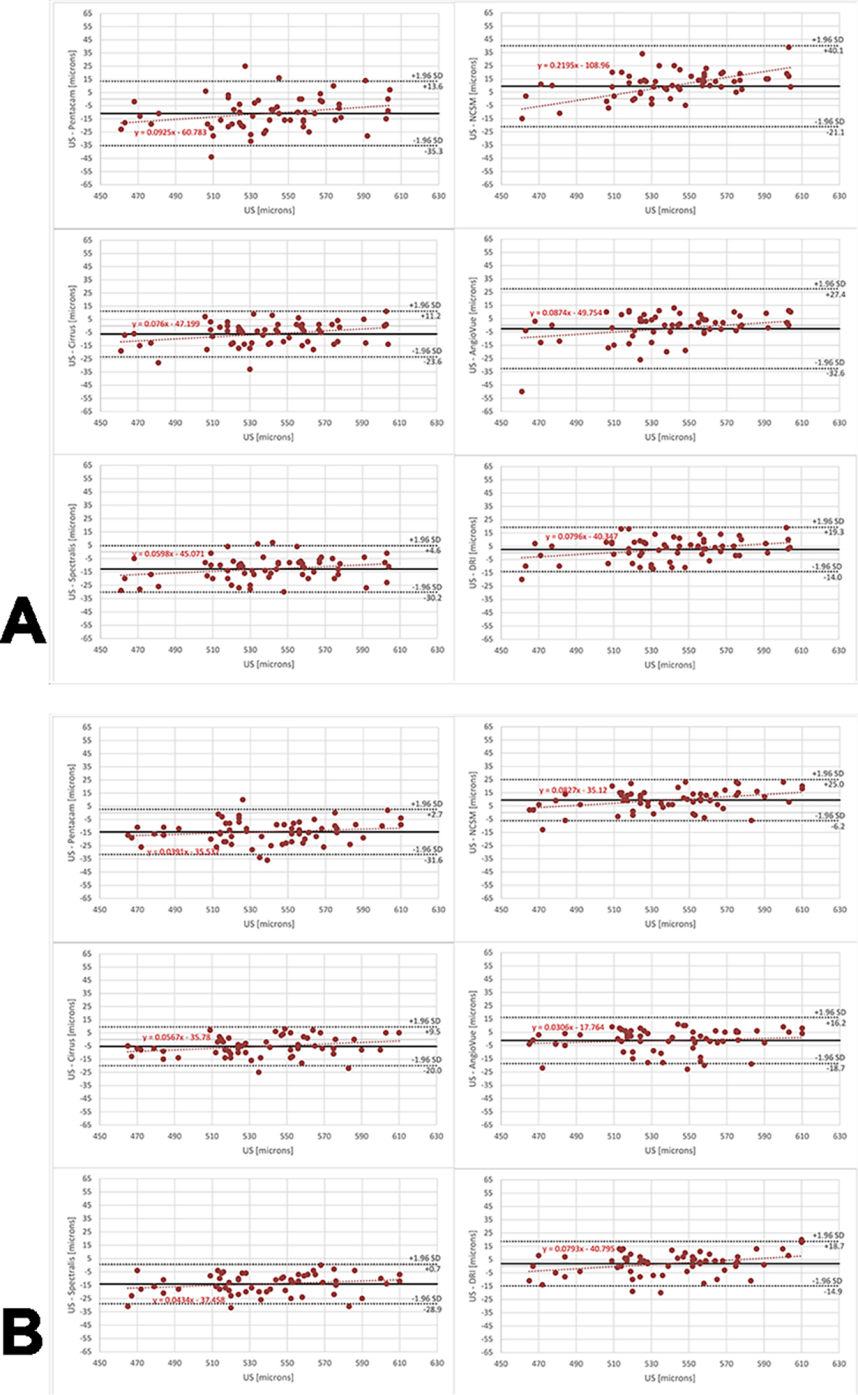
Bland-Altman analysis of all measured data of the left eye (A) and right eye (B) compared to US pachymetry. The results suggest that the Pentacam and the Spectralis SDOCT overestimate CCT with respect to the ultra-sound device. Further, the DRI SSOCT also slight overestimates the values, with respect to US measurements. The results further suggest a good agreement of both the Cirrus SDOCT and the AngioVue with US pachymetry measurements. On one hand, the NCSM device corresponds well with US pachymetry for lower values, but on the other hand shows underestimation for higher CCT values.

All devices were compared to the US pachymetry device, because US pachymetry has formerly been considered the gold standard in CCT measurement, and most ophthalmologists are familiar with this device. The 95% limit of agreement (LoA) of the differences between devices was (OS) -31.6μm to +2.7μm, -6.2μm to +25.0μm, -20.0μm to +9.5μm, -18.7μm to +16.2μm, -28.9μm to +0.7μm, and -14.8μm to 18.7μm; and (OD) -35.3μm to +13.6μm, -21.1μm to +40.1μm, -23.6μm to +11.2μm, -32.6μm to +27.4μm, -30.2μm to +4.6μm, and -14.0μm to 19.3μm, for US minus Pentacam, US minus NSCM, US minus Cirrus, US minus AngioVue, US minus Spectralis, and US minus DRI. Further, Bland-Altman difference plots were used to represent these comparisons (Fig 3), because they argue that any two methods that are designed to measure the same parameter should have good correlation.

### Mean absolute deviation

Deviation of the individual devices from the mean CCT value of each eye was (OS/OD) for Pentacam 11.3±5.9/10.6±7.3 μm μm(SD), Topcon NCSM 12.8±5.0/14.9±9.4 μm μm(SD), Cirrus OCT 4.2±3.2/5.7±4.6 μm μm(SD), AngioVue OCT 5.1±3.4/5.9±10.3 μm μm(SD), Spectralis OCT 10.7±5.2/10.4±4.8 μm μm(SD), Topcon DRI OCT 6.0±3.9/6.2±4.9 μm μm(SD), and US pachymetry 4.8±4.1/5.7±4.6 μm μm(SD).

## Discussion

This study compared an unprecedented number of devices for central corneal thickness measurements. The aim was to define the lowest deviation from the overall mean CCT value.

CCT measurement results in this study were in good agreement with previous reports. [23, 24] With respect to the inter-eye correlation, a considerably large inter-device variation was found. While, the results of this study indicate that Pentacam and spectral OCT are quite consistent [25], the results of all other OCT-based devices in this study seem to rather agree with the ultrasound device.

In contrast, previous studies have found that OCT devices underestimated CCT compared to US pachymetry.[26, 27] But since these studies were performed with a time-domain OCT and not with spectral- or swept-source technologies, their comparability to our study is limited. Swept-source OCT measured slightly higher values than US pachymetry, which, however, is in agreement with prior findings.[28] Chaudhry et al. presented CCT data from NCSM, which compared well with US pachymetry. However, they used a different generation of device than this study did and performed their study on a smaller population. [29] Additionally, to compare each device’s measurement results with the US value, measurement deviation was analyzed by calculating mean absolute deviation from mean CCT values per eye. Ultrasound, DRI, AngioVue and the Cirrus were all close to the mean, the other three devices (Pentacam, NCSM, and Spectralis) showed more than doubled deviation. Thus, a new insight of this study was that Cirrus OCT showed the lowest deviation from the mean CCT value.

This study could be limited because no repeated measurements were performed to assess device precision. However, repeatability of CCT measurements has already been reported earlier. [8, 10, 24, 30, 31] Furthermore, it has been discussed in literature that better axial resolutions might positively influence the accuracy of cornea thickness measurement, mainly because the individual layers (such as for example Bowman’s membrane) inside the cornea can be distinguished more clearly [32]. Hence, comparisons of CCT measurement devices may inherently be limited because of the differences in their respective resolutions.

Another limitation may be that this study did not compare corneal pachymetry values, because the US device does not provide thinnest pachymetry, as the decentered point cannot easily be detected by punctual measurements. Still it was crucial to include the US device, as clinical practice has traditionally been relying on that method for decision-making. Furthermore, the results may not be applicable to patients with pathological corneas or eyes that have had prior corneal surgery because of potentially change in biomechanical variables and corneal shape. Finally, this study demonstrates that the CCT measurement by OCT showed slightly superior results compared to the other measurement methods.

## Supporting information

S1 Table Central corneal thickness measurement data are included. (PDF)
