# Supplementary material for: Interdevice variability of central corneal thickness measurement
Source: PLoS One. 2018 Sep 13;13(9):e0203884. doi: 10.1371/journal.pone.0203884 (PMC6136793; doi:10.1371/journal.pone.0203884)
Supplement: S1 Table — (PDF) [file pone.0203884.s001.pdf]

S1 table 1 **Central Corneal Thickness CCT**

subject	Corneal thickness measurements OD								Corneal thick
	Pentacam	Topcon	ultrasound	OCTs					Pentacam
No	Pentacam	NCSM	US	Cirrus	Avanti	Spectralis	DRI		Pentacam
1	484	476	461	480	511	490	481		477
2	562	535	530	563	536	555	539		554
3	564	553	548	560	567	578	559		569
4	526	514	507	525	524	525	515		518
5	586	563	577	589	578	590	579		595
6	620	577	592	605	594	612	592		609
7	577	576	591	586	582	595	584		607
8	591	570	575	589	579	595	577		585
9	534	519	524	528	521	535	526		516
10	542	520	524	541	550	551	535		544
11	557	547	557	557	552	564	558		563
12	557	534	530	547	526	558	541		495
13	531	500	509	506	511	519	502		538
14	579	545	558	573	563	575	555		581
15	565	541	555	554	553	551	550		569
16	550	517	542	534	529	535	530		559
17	529	521	521	534	525	528	513		540
18	568	548	567	566	570	575	566		580
19	569	536	559	563	555	571	556		572
20	539	506	518	518	532	538	515		538
21	575	551	564	582	562	579	560		591
22	550	535	544	547	544	557	544		553
23	481	461	463	470	467	483	473		482
24	549	531	541	554	541	557	552		569
25	492	492	481	509	493	507	491		501
26	529	523	545	544	536	553	534		571
27	539	521	520	534	528	545	528		538

28	562	531	538	541	558	552	534		575
29	484	460	471	486	484	499	473		486
30	592	571	578	579	578	587	568		595
31	547	512	526	531	518	537	518		531
32	515	501	518	522	508	532	518		525
33	502	514	527	541	524	543	520		542
34	500	508	506	499	496	514	498		504
35	603	564	603	592	592	604	593		610
36	612	586	603	602	603	626	600		619
37	570	548	568	573	566	572	563		568
38	556	540	540	554	545	559	547		574
39	563	529	537	544	537	541	531		566
40	553	489	509	512	511	510	501		529
41	556	538	545	550	544	554	540		557
42	575	541	558	557	554	567	550		576
43	597	595	604	618	594	615	600		601
44	470	552	468	474	465	473	461		481
45	544	522	531	544	536	545	543		528
46	535	509	534	538	523	528	532		539
47	610	583	602	602	600	610	583		614
48	538	508	510	518	525	530	511		529
49	586	551	561	573	564	581	567		583
50	545	491	525	528	522	534	525		532
51	564	561	574	573	564	579	569		575
52	567	534	551	560	552	559	545		579
53	563	558	567	570	564	573	553		561
54	530	494	514	515	506	524	496		530
55	556	542	556	550	548	564	542		564
56	517	501	518	515	507	514	500		514
57	535	519	532	523	526	549	518		527
58	528	511	524	525	519	534	515		526
59	496	467	477	490	477	494	472		495
60	574	539	558	560	564	569	548		572

61	581	558	577	573	570	590	564		588

kness measurements OS

Topcon	ultrasound	OCTs			
NCSM	US	Cirrus	Avanti	Spectralis	DRI
485	472	480	494	490	486
532	536	550	554	562	543
550	564	557	558	568	560
506	515	515	525	521	502
570	586	586	580	590	573
578	590	598	593	615	589
589	583	605	602	620	594
552	575	586	569	597	575
511	526	531	520	532	522
522	520	534	535	543	539
547	556	560	554	568	554
470	479	486	483	495	484
506	516	518	510	521	510
562	558	576	578	582	571
546	556	563	570	563	553
532	531	547	540	548	538
523	528	531	546	534	526
565	568	563	570	568	564
539	549	541	572	568	553
515	512	522	512	528	513
554	576	576	571	588	573
525	544	538	533	555	538
463	465	470	469	496	476
528	535	560	546	555	555
490	484	499	489	505	492
536	547	544	537	557	540
519	520	531	530	552	530

533	539	541	541	559	539
465	467	480	468	490	467
552	569	576	564	582	572
513	524	528	526	546	521
508	517	525	514	534	511
520	528	538	524	548	525
486	492	506	489	510	496
577	600	608	591	605	587
590	610	605	606	614	592
544	552	560	552	577	552
553	552	566	559	564	548
535	541	547	540	559	529
489	509	502	500	517	505
525	548	544	538	557	538
552	561	560	563	568	552
595	603	598	598	610	595
464	470	477	467	474	462
517	524	534	522	535	532
504	517	531	516	534	513
592	610	605	602	610	590
501	514	515	516	532	514
559	565	570	568	572	575
497	519	528	517	532	523
562	575	582	570	578	573
546	559	557	559	567	555
555	553	566	556	566	548
499	514	512	507	524	502
538	552	547	547	563	545
498	513	515	505	517	500
505	519	518	513	538	510
511	524	531	516	534	517
470	484	493	480	495	477
542	556	560	573	563	545

	561	576	576	577	591	569
